# Fcµ Receptor Promotes the Survival and Activation of Marginal Zone B Cells and Protects Mice against Bacterial Sepsis

**DOI:** 10.3389/fimmu.2018.00160

**Published:** 2018-02-05

**Authors:** Jun Liu, Hanying Zhu, Jiawen Qian, Ermeng Xiong, Lumin Zhang, Yan-Qing Wang, Yiwei Chu, Hiromi Kubagawa, Takeshi Tsubata, Ji-Yang Wang

**Affiliations:** ^1^Department of Immunology, School of Basic Medical Sciences, Fudan University, Shanghai, China; ^2^Department of Integrative Medicine and Neurobiology, School of Basic Medical Sciences; Institute of Acupuncture and Moxibustion, Fudan Institutes of Integrative Medicine, Fudan University, Shanghai, China; ^3^Deutsches Rheuma-Forschungszentrum Berlin, Berlin, Germany; ^4^Department of Immunology, Medical Research Institute, Tokyo Medical and Dental University, Tokyo, Japan

**Keywords:** marginal zone B cell, Fcµ receptor, tonic BCR signal, apoptosis, humoral immune response

## Abstract

The marginal zone B cells (MZB) are located at the interface between the circulation and lymphoid tissue and as a gatekeeper play important roles in both innate and adaptive immune responses. We have previously found that MZB are significantly reduced in mice deficient in the IgM Fc receptor (FcμR) but how FcμR regulates the development and function of MZB remains unknown. In this study, we found that both marginal zone precursor (MZP) and MZB were decreased in *FcμR*^−/−^ mice. The reduction of MZP and MZB was not due to impaired proliferation of these cells but rather due to their increased death. Further analysis revealed that *FcμR*^−/−^ MZB had reduced tonic BCR signal, as evidenced by their decreased levels of phosphorylated SYK and AKT relative to WT MZB. MZB in *FcμR*^−/−^ mice responded poorly to LPS *in vivo* when compared with MZB in WT mice. Consistent with the reduced proportion of MZB and their impaired response to LPS, antibody production against the type 1 T-independent Ag, NP-LPS, was significantly reduced in *FcμR*^−/−^ mice. Moreover, *FcμR*^−/−^ mice were highly susceptible to *Citrobacter rodentium*-induced sepsis. These results reveal a critical role for FcμR in the survival and activation of MZB and in protection against acute bacterial infection.

## Introduction

Mature B lymphocytes can be divided into B1, follicular B (FOB), and marginal zone B cells (MZB) (1). MZB are distinguished from FOB in that they are non-circulating mature B cells and located as a gatekeeper at the interface between the circulation and lymphoid tissues. MZB can efficiently bind to the blood-borne antigens (Ag) and rapidly differentiate into antibody-secreting plasmablasts in the presence of costimulatory signals from innate or adaptive immune cells (2). MZB can also transport blood-borne Ag to B cell follicles to initiate adaptive responses. Therefore, this innate-like B cell subset helps to bridge the spatiotemporal gap between the innate immunity and a primary, T-cell dependent, adaptive antibody response (3). Besides, unlike FOB that primarily express monoreactive BCR, MZB express polyreactive BCR that can recognize multiple microbial molecular patterns (4–6).

Marginal zone B cells are thought to derive from bone marrow and not fully formed until 2–3 weeks after birth in rodents (7). Currently, it is considered that MZB mainly develop through the following stages: transitional B1 (T1), transitional B2 (T2), MZ precursor (MZP), and finally MZB (1, 8–10). MZB fate decision depends on BCR (8), Notch2 (11, 12), and BAFF receptor (BAFFR) signaling (13). BCR signaling might be integrated with BAFFR and Notch2 signaling during the commitment to MZB or FOB lineage (14–17).

Marginal zone B cells and FOB exhibit different gene expression profiles (18, 19), which contribute to their differential localization and function. MZB have an IgM^high^IgD^low^CD21^high^CD23^−^CD1d^high^ phenotype (20), which is distinct from FOB that are IgM^low^IgD^high^CD21^low^CD23^high^CD1d^−^. MZB also express higher levels of MHC class II, CD80, and CD86 compared with FOB (6, 21). Moreover, our previous study shows that both MZB and FOB express Fcμ receptor (FcμR) (22), a *bona fide* FcμR specific for pentameric IgM (23, 24). FcμR is predominantly expressed by B cells in mice and B, T, and NK cells in humans (22–27). In addition, FcμR has been shown to regulate the activation of monocytes, macrophages, and granulocytes (28), the differentiation and activation of dendritic cells (29, 30), and the function of human T and NK cells (31). Both Honjo et al. (25) and Ouchida et al. (22) demonstrated that mice lacking FcμR exhibited reduced MZB population, elevated serum IgM levels, impaired humoral immune responses to a T-dependent Ag and autoantibody production. More recently, Nguyen et al. generated B cell-specific FcμR-deficient mice and found that FcμR constrained surface BCR expression and its absence in B cells resulted in elevated BCR levels and enhanced tonic BCR signaling (27). They also found increased numbers of B-1 cells in the spleen, which showed enhanced activation and differentiation into antibody-secreting cells (27). Consistent with previous studies (22, 25), mice with B cell-specific FcμR deficiency produced autoantibodies and exhibited reduced antiviral IgG production (27, 32). We have recently found that FcμR interacts and cooperates with the BCR to promote the survival of splenic B cells in mice (33). Clinically, FcμR is highly expressed on B cell chronic lymphocytic leukemia (23, 34–36), which suggests a role for FcμR in promoting the survival of such malignant cells. Intriguingly, MZB are significantly decreased in the spleen of *FcμR*^−/−^ mice (22, 25, 37). It has been suggested that reduced numbers of MZB in *FcμR*^−/−^ mice resulted from their rapid differentiation into plasma cells (37) but how FcμR regulates MZB development and function remains unknown.

In this study, we have analyzed the role of FcμR in the development, survival, and activation of MZB. We found a reduction of both MZP and MZB in *FcμR*^−/−^ mice from 6 weeks of age when compared with WT mice. The reduction of MZB in *FcμR*^−/−^ mice was associated with an increased death of these cells. Further analysis revealed that *FcμR*^−/−^ MZB had reduced tonic BCR signal. *FcμR*^−/−^ MZB had impaired response *in vivo* to LPS and consistently the mutant mice exhibited a severe impairment in antibody production against the T-independent (T-I) Ag NP-LPS. *FcμR*^−/−^ mice were also highly susceptible to bacterial sepsis. These results demonstrate a critical role for FcμR in tonic BCR signaling in MZB and their survival and LPS response, and in protection against acute bacterial infection.

## Materials and Methods

### Mice

WT and *FcμR*^−/−^ mice (22) were maintained in specific pathogen-free conditions in the Department of Laboratory Animal Science, Fudan University. All animal experiments were approved by the Animal Committee of the School of Basic Medical Sciences, Fudan University. Mice under 12 weeks of age were used for all the experiments.

### Flow Cytometry (FACS)

Mouse spleen or bone marrow were obtained from euthanized mice and single-cell suspension was prepared after lysing the red blood cells. For analyzing FcμR expression in various B-cell subpopulations, cells were first incubated with aggregated human IgG (prepared by incubating the antibody in a 60°C water bath for 30 min and then chilling in ice water) to block all FcγRs and then stained with biotin-MM3 anti-FcμR mAb (25, 37), followed by PE-labeled streptavidin. For staining with other specific mAbs, cells were first incubated with rat IgG2b anti-mouse CD16/CD32 monoclonal antibody (clone 2.4G2; BD Biosciences) to block FcγR and then stained with FITC-, PE-, allophyocyanin-, PerCP-Cyanine5.5-, or PE/Cy7-conjugated antibodies against various surface molecules expressed during B cell development and differentiation (38). For intracellular staining, cells were fixed in 2% paraformaldehyde at 37°C for 10 min and then blocked with rat IgG_2b_ anti-mouse CD16/CD32 for 20 min. The cells were then stained with antibodies against phosphorylated SYK (pSYK) (pY348) and AKY (pS473) (diluted in 1× PBS containing 10% FBS and 0.1% Triton X-100) for 20 min in the dark, and washed with 1× PBS containing 4% FBS. The stained cells were analyzed on a FACSVerse flow cytometer (BD Biosciences) using the FACSuite software. Antibodies used in this study are listed in Table S1 in Supplementary Material.

### Immunofluorescence

Whole spleens were frozen in Tissue-Tek OCT compound (SAKURA). Cryosections (8-µm thick) were mounted onto slides, air dried for 30 min, fixed in ice-cold acetone/methanol (1:1) for 10 min, rehydrated in PBS, and blocked for 60 min with blocking buffer (PBS containing 1% BSA and 0.1% Tween 20), followed by three 5-min washes in PBS. Sections were stained with FITC-conjugated rat anti-mouse B220 (BD Biosciences) and biotinylated rat anti-mouse metallophilic macrophage (MOMA-1, MCA947G; Serotec) diluted in blocking buffer, incubated at 4^°^C overnight, and then stained with Cy3-labeled streptavidin at room temperature for 1 h, followed by three 5-min washes in PBS. The slides were finally mounted with low fluorescent glycerol and coverslip protection, and observed with a Nikon Eclipse Ti-S inverted microscope.

### EdU Incorporation Assay

EdU incorporation was performed using a Click-iT EdU Alexa Fluor kit (C10424, ThermoFisher) according to the manufacturer’s instruction. Briefly, WT and *FcμR*^−/−^ mice (12 weeks old) were injected intraperitoneal (i.p.) with 200 µl of (1 mg/ml) EdU. EdU incorporation was analyzed 24 h later by FACS.

### Immunization and ELISA Assay

These experiments were performed essentially as described elsewhere (38, 39).

### Infection Experiment

*Citrobacter rodentium* suspended in 200 µl of PBS or PBS alone were injected into the tail vein of WT and *FcμR*^−/−^ mice as described previously (40) and monitored for their survival.

### Statistical Analysis

Statistical significance was assessed by an unpaired t-test or log-rank test (*p < 0.05; **p < 0.01; ***p < 0.005).

## Results

### Decreased Numbers of MZB and Reduced MZB Area in the Spleen of *FcμR*^−/−^ Mice

**Figure 1:**
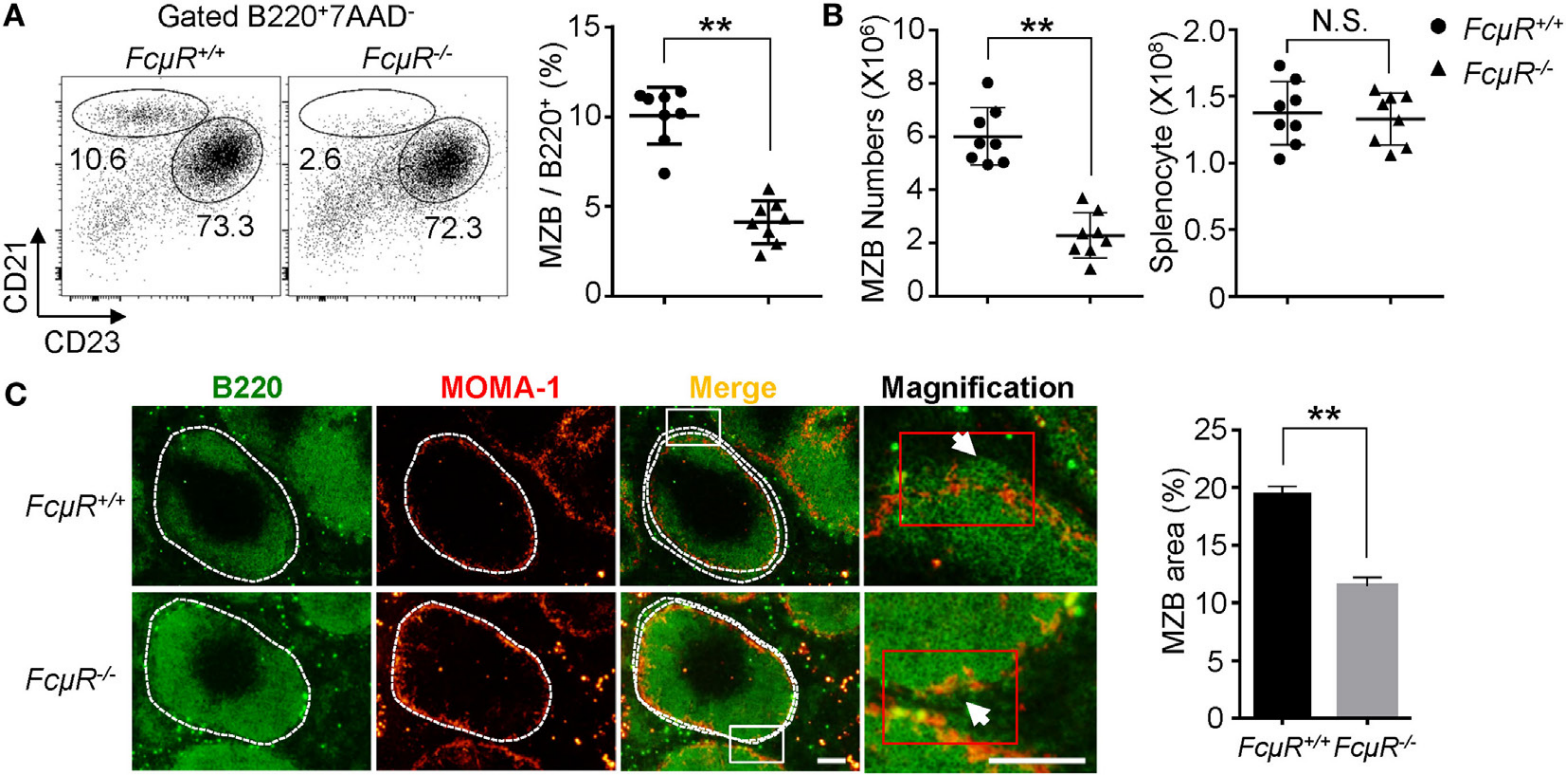
Decreased numbers of marginal zone B cells (MZB) and reduced MZB area in the spleen of *FcμR*^−/−^ mice. **(A)** Reduced proportion of the B220^+^CD21^high^CD23^low^ MZB in *FcμR*^−/−^ mice. Left, representative FACS profiles; right, summary of eight pairs of WT and *FcμR*^−/−^ mice. **(B)** Absolute numbers of MZB (left) and total spleen cells (right). The results of eight pairs of mice are shown. **(C)** Spleen sections of WT and *FcμR*^−/−^ mice were stained for B220 (green) and mouse metallophilic macrophage (MOMA-1) (red). Scale bars, 100 µm. The proportion of MZB area was calculated by dividing the B220^+^ area outside the MOMA-1^+^ ring by the total B220^+^ area (*n* = 32; four MZB areas per section, four sections per mouse, two mice per group) using the Image J software. Representative staining results (left) and the mean MZB area (%) ± SD are shown. *p < 0.05; **p < 0.01.

We previously found the reduced MZB compartment in *FcμR*^−/−^ mice when compared with WT mice (22, 25). To confirm this result and further clarify the reduction in the absolute numbers of MZB, we analyzed eight pairs of WT and *FcμR*^−/−^ mice at 8–12 weeks of age. FACS analysis of CD23 and CD21 expression confirmed the significant reduction in the proportion of the CD21^high^CD23^−^ MZB (Figure 1A). The absolute numbers of MZB in the spleen of each mouse were also greatly reduced in *FcμR*^−/−^ mice (Figure 1B, left panel) while the total numbers of spleen cells were not different between WT and *FcμR*^−/−^ mice (Figure 1B, right panel). To further verify the reduction of MZB, we stained spleen sections with MOMA-1, which is expressed by the metallophilic macrophages adjacent to the MZ of the spleen, and B220. As shown in Figure 1C, the spleen of *FcμR*^−/−^ mice contained a significantly reduced MZB area compared with that of WT mice. These results demonstrate that the absence of FcμR results in reduction of MZB cell numbers, consistent with the previous findings (22, 25, 37).

### MZB Development in *FcμR*^−/−^ Mice

**Figure 2:**
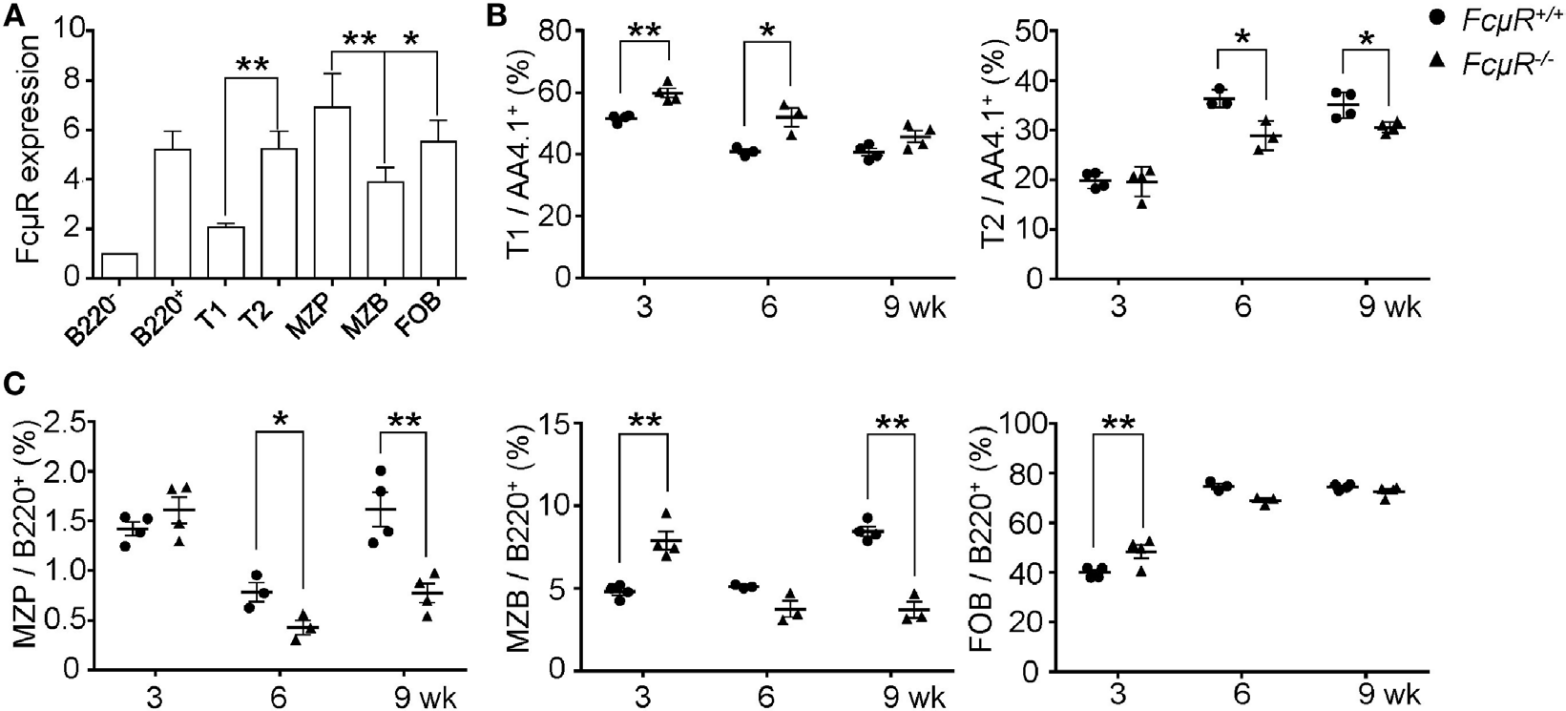
Marginal zone B cells (MZB) development in 3-, 6-, and 9-week-old WT and *FcμR*^−/−^ mice. **(A)** Fc receptor (FcμR) expression at different stages of MZB development. MFI ± SD of the results from four 8- to 10-week-old mice are shown. MFI of the B220^−^ cells was set to 1. **(B)** The proportion of T1 and T2 in 3-, 6-, and 9-week-old WT and *FcμR*^−/−^ mice (*n* = 3 or 4). **(C)** The proportion of marginal zone precursor (MZP), MZB, and follicular B (FOB) in 3-, 6-, and 9-week-old WT and *FcμR*^−/−^ mice (*n* = 3 or 4). The gating strategy is shown in Figure S1 in Supplementary Material. *p < 0.05; **p < 0.01.

To explore the mechanism for the reduced MZB population in *FcμR*^−/−^ mice, we first analyzed FcμR expression at different stages of MZB development by FACS. The gating strategy is shown in Figure S1 in Supplementary Material. FcμR expression was found to be low at T1, increased at T2, and further upregulated at MZ precursor (MZP), and then slightly reduced at MZB stage (Figure 2A). To explore the role of FcμR in MZB development, we next analyzed the proportion of T1, T2, MZP, and MZB in mice of 3, 6, and 9 weeks of age during which period MZB are formed. In WT mice, T1 decreased and T2 increased at 6 weeks of age compared with 3 weeks of age, and then both population remained unchanged during 6–9 weeks of age (Figure 2B). Compared with WT mice, *FcμR*^−/−^ mice had a higher proportion of T1 at 3 weeks of age, which continued decreasing at 6 and 9 weeks of age accompanied by a gradual increase of T2 at 6 and 9 weeks of age (Figure 2B). These observations suggest that the decrease of T1 and the increase of T2 were both delayed in *FcμR*^−/−^ mice when compared with WT mice. In WT mice, the proportion of MZP was transiently decreased at 6 weeks of age and then increased at 9 weeks of age (Figure 2C, left panel), accompanied by a similar increase of MZB at 9 weeks of age (Figure 2C, middle panel). While the proportion of MZP was similarly decreased at 6 weeks of age in *FcμR*^−/−^ mice, it only slightly increased at 9 weeks of age (Figure 2C, left panel) and was not accompanied by an increase of MZB at 9 weeks of age (Figure 2C, middle panel). Therefore, the proportion of MZP and MZB both failed to recover at 9 weeks of age in *FcμR*^−/−^ mice. Collectively, absence of FcμR resulted in a partial reduction of T2 and a severe reduction of MZP and MZB at 9 weeks of age. In contrast, the proportion of FOB was quite similar between WT and *FcμR*^−/−^ mice at 3, 6, and 9 weeks of age (Figure 2C, right panel), indicating that FcμR specifically affected MZB cell development.

### MZB in *FcμR*^−/−^ Mice Exhibit Increased Cell Death and Turnover *In Vivo*

**Figure 3:**
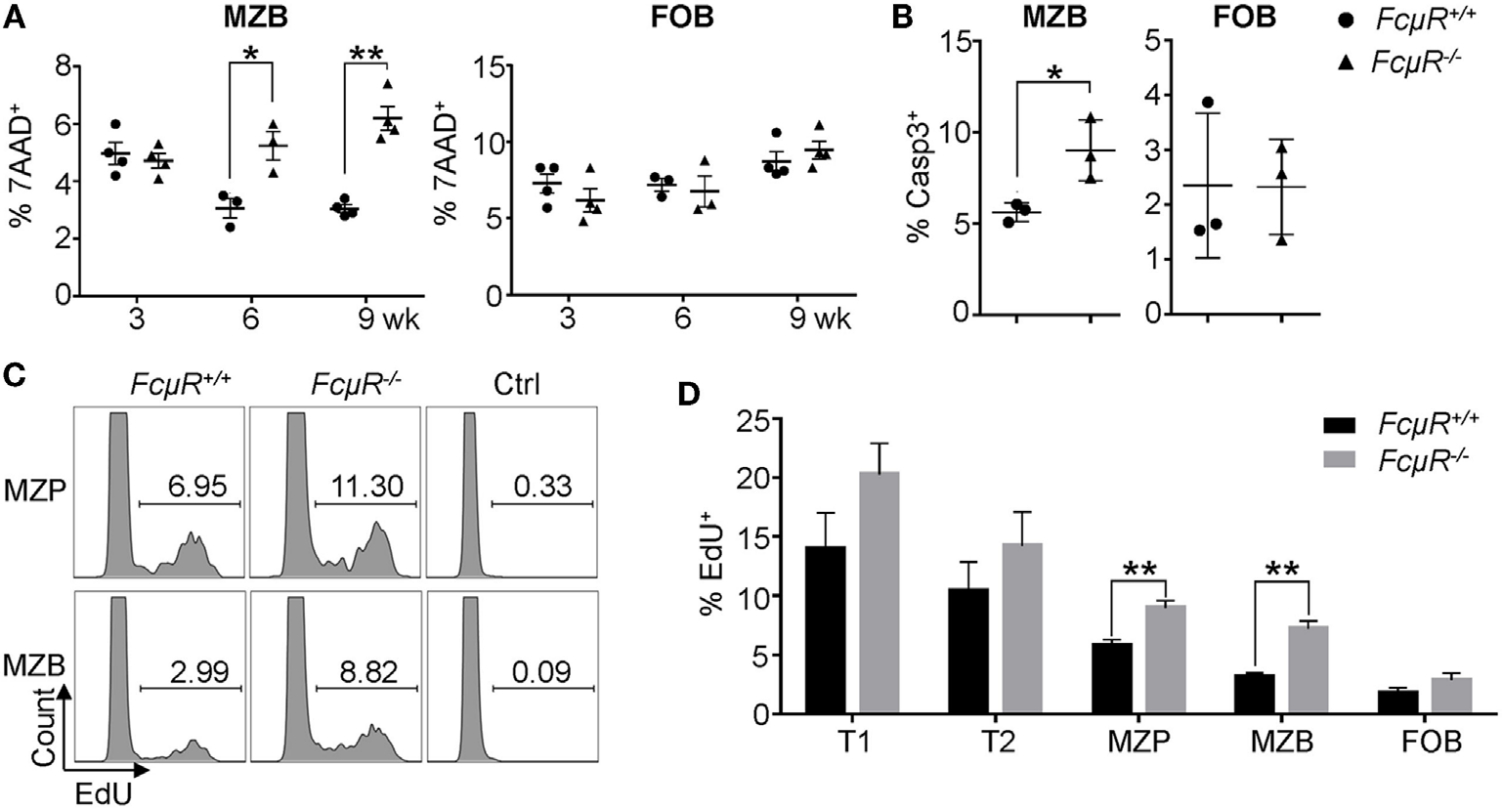
Increased death and turnover in marginal zone B cells (MZB) of *FcμR*^−/−^ mice. **(A)** The proportion of 7AAD^+^ dead cells in gated MZB and follicular B (FOB) in the spleen. **(B)** Increased proportion of Casp3^+^ MZB in *FcμR*^−/−^ mice. **(C)** Increased EdU^+^ marginal zone precursor (MZP) and MZB population in *FcμR*^−/−^ mice. **(D)** Proportion of EdU^+^ cells at each stage of MZB development. Mean ± SD of the results from three to four mice are shown. For panels **(B–D)**, 10- to 12 week-old mice were analyzed. *p < 0.05; **p < 0.01.

We next sought to investigate the mechanism for the observed reduction in MZP and MZB in *FcμR*^−/−^ mice. The decrease in MZB could be due to their increased death, decreased expansion, developmental arrest, or accelerated differentiation into plasma cells (37). To explore these possibilities, we first analyzed death of freshly isolated MZB and FOB. As shown in Figure S2 in Supplementary Material and Figure 3A (left panel), MZB in 6- and 9-week-old *FcμR*^−/−^ mice exhibited significantly increased percentages of the 7AAD^+^ dead cells compared with MZB in WT mice. Consistently, the mutant MZB contained a greater proportion of cells positive for active caspase-3 (Figure 3B, left panel). In contrast, FOB in WT and *FcμR*^−/−^ mice contained a similar percentage of the dead cells (Figure 3A, right panel) and cells positive for active caspase-3 (Figure 3B, right panel). These observations indicate that *FcμR*^−/−^ MZB had increased spontaneous apoptosis *in vivo* compared with WT MZB.

We then analyzed the proliferation *in vivo* of cells at different stages of MZB development by EdU incorporation assay. Mice were injected with EdU and 1 day later the proportion of EdU^+^ cells was analyzed. Both MZP and MZB in *FcμR*^−/−^ mice contained a higher proportion of the EdU^+^ cells compared with those in WT mice (Figure 3C). In addition, T1 and T2 cells in *FcμR*^−/−^ mice also contained a slightly increased proportion of the EdU^+^ cells compared with those in WT mice but the difference did not reach statistical significance (Figure 3D). In contrast, FOB in WT and *FcμR*^−/−^ mice contained a similar proportion of the EdU^+^ cells. These results implicated a higher rate of turnover for cells at each stage of MZB development in *FcμR*^−/−^ mice, possibly to compensate for the increased death.

### Elevated IgD and MHC Class II Expression in MZB Cells of *FcμR*^−/−^ Mice

**Figure 4:**
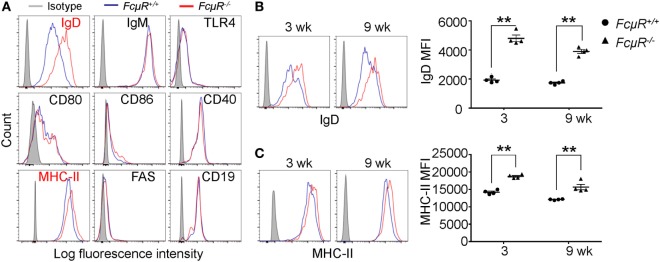
Elevated IgD and MHC class II expression in *FcμR*^−/−^ marginal zone B cells (MZB) cells. **(A)** Expression of IgD, IgM, TLR4, CD80, CD86, CD40, MHC-II, FAS, and CD19 in WT (blue) and *FcμR*^−/−^ (red) MZB. **(B)** IgD and **(C)** MHC-II expression in MZB from 3- and 9-week-old WT and *FcμR*^−/−^ mice. Left panels, representative FACS profiles; right panels, the results of four WT and *FcμR*^−/−^ mice. *p < 0.05; **p < 0.01.

To gain insight into the functional differences between MZB in WT and *FcμR*^−/−^ mice, we compared the expression of various cell surface molecules that are known to play important roles in B cell function. We found that MZB in *FcμR*^−/−^ mice expressed higher levels of IgD and MHC class II than did MZB in WT mice (Figure 4A). The levels of IgM, TLR4, CD80, CD86, CD40, FAS, and CD19 were not different between WT and *FcμR*^−/−^ MZB (Figure 4A). The increased expression of IgD and MHC class II was observed in *FcμR*^−/−^ mice at both 3 and 9 weeks of age (Figures 4B,C).

### Reduced Tonic BCR Signaling in *FcμR*^−/−^ MZB

**Figure 5:**
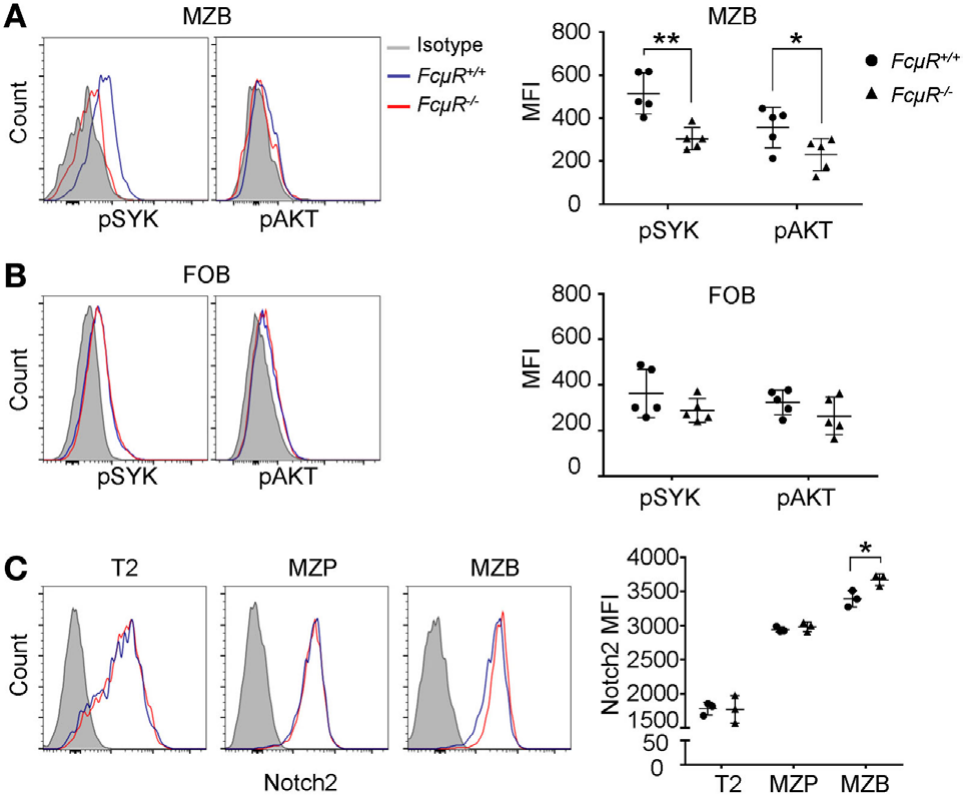
Reduced tonic BCR signaling and increased Notch2 expression in *FcμR*^−/−^ marginal zone B cells (MZB). Reduced levels of phosphorylated SYK (pSYK) and AKT in MZB **(A)** but not follicular B (FOB) **(B)** of naïve *FcμR*^−/−^ mice. Left panels, representative FACS profiles; right panels, quantitation of MFI of pSYK (pY348) and AKT (pS473) in MZB from five pairs of WT and *FcμR*^−/−^ mice. **(C)** Elevated Notch2 expression in *FcμR*^−/−^ MZB. Left panel, representative FACS profiles; right panel, results of three pairs of WT and *FcμR*^−/−^ mice. *p < 0.05; **p < 0.01.

The strength of BCR and Notch2 signaling is important for MZB development. Tonic BCR signaling triggers phosphorylation of SYK and its downstream targets, including ERK and AKT kinases. Intracellular staining revealed significant reduction in the levels of pSYK and pAKT in *FcμR*^−/−^ MZB when compared with WT MZB (Figure 5A; Figure S3 in Supplementary Material). These results suggest that MZB in *FcμR*^−/−^ mice have reduced tonic BCR signal, consistent with their decreased survival (Figure 3A). In contrast, pSYK and pAKT levels were not significantly different between WT and *FcμR*^−/−^ FOB (Figure 5B). In addition, we found normal Notch2 expression in *FcμR*^−/−^ T2 and MZP and a moderately increased Notch2 level in *FcμR*^−/−^ MZB relative to WT MZB (Figure 5C). Notch2 expression has been shown to be critical for the cell fate determination of MZB (11, 12). These observations suggest that the reduction of MZP and MZB in *FcμR*^−/−^ mice is unlikely due to an impaired MZB fate decision but rather due to reduced tonic BCR signaling and increased cell death.

### Decreased Antibody Production against Type 1 T-I Ag in *FcμR*^−/−^ Mice

**Figure 6:**
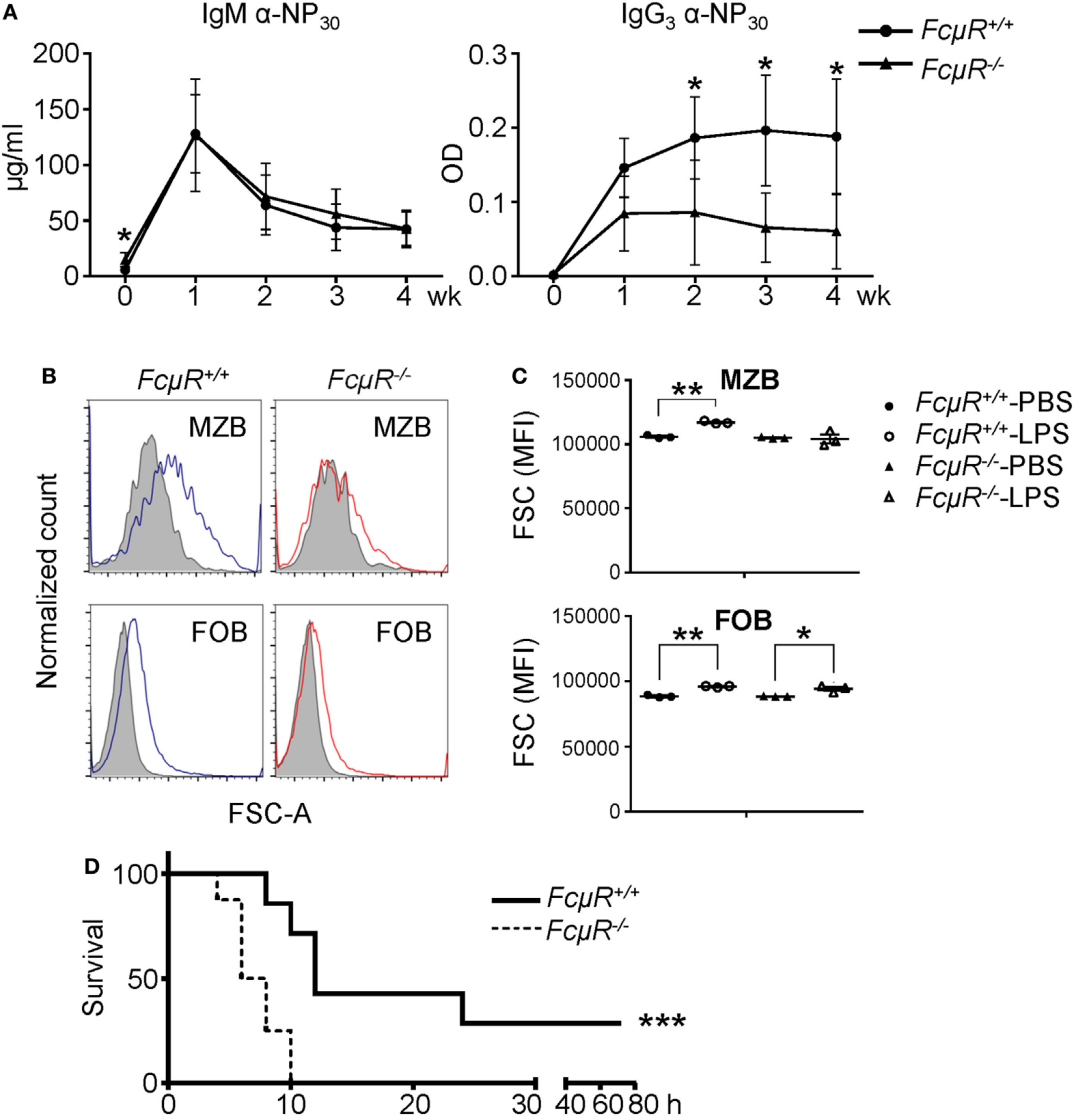
*FcμR*^−/−^ mice are impaired in response to LPS and highly susceptible to *Citrobacter rodentium* infection. **(A)** Antibody production to a TI antigen, NP-LPS. Eight pairs of WT and *FcμR*^−/−^ mice were immunized i.v. with 10 µg of NP-LPS and analyzed for the production of NP-specific IgM (left) and IgG_3_ (right) antibodies in the serum at 1–4 weeks after immunization. The mean values of WT and *FcμR*^−/−^ mice at the indicated time points are shown. **(B,C)** Marginal zone B cells (MZB) in *FcμR*^−/−^ mice responded poorly to LPS administration. WT and *FcμR*^−/−^ mice were injected i.v. with 20 µg LPS or PBS as a control, and the cell size (FSC) of MZB and follicular B (FOB) was analyzed 24 h later. **(B)** FSC of MZB and FOB in WT (blue lines) and *FcμR*^−/−^ (red lines) mice injected with LPS. Shaded areas, FSC of MZB and FOB in mice injected with PBS. **(C)** Summary of three mice per group. *p < 0.05; **p < 0.01 (unpaired *t*-test). **(D)** *FcμR*^−/−^ mice are highly susceptible to acute bacterial infection. Eight pairs of WT and *FcμR*^−/−^ mice were injected i.v. with *C. rodentium* (6 × 10^8^ CFU) or PBS as a control and monitored for their survival. Kaplan–Meier survival plot for WT (solid line) and *FcμR*^−/−^ (dotted line) mice are shown. ***p < 0.005 (log-rank test).

Marginal zone B cells and B1 cells are thought to be the major sources that produce antibodies in response to T-I and self Ags. To explore the function of MZB in *FcμR*^−/−^ mice, we analyzed antibody production against type 1 T-I Ag NP-LPS. Consistent with the reduced MZB population, the production of NP-specific IgG_3_ antibody was significantly reduced in *FcμR*^−/−^ mice (Figure 6A, right panel). The production of NP-specific IgM seemed unaffected (Figure 6A, left panel). However, our previous study has revealed that the basal serum IgM levels are elevated in *FcμR*^−/−^ mice possibly due to the lack of FcμR-mediated IgM binding (22). Therefore, the seemingly normal production of the NP-specific IgM in *FcμR*^−/−^ mice could be due to the lack of FcμR-mediated absorption of these antibodies. To analyze MZB and FOB activation *in vivo* in WT and *FcμR*^−/−^ mice after the immunization, we compared their cell sizes as described by others (41). After i.v. injection of LPS, WT MZB increased their cell sizes significantly whereas *FcμR*^−/−^ MZB did not (Figure 6B, upper left vs. right panels). By contrast, FOB in both groups of mice increased their cell sizes significantly (Figure 6B low panels). The results of three pairs of WT and *FcμR*^−/−^ mice are summarized in Figure 6C. These results demonstrate that MZB in *FcμR*^−/−^ mice responded poorly to LPS than did MZB in WT mice and suggest that FcμR is required for MZB to produce antibodies against NP-LPS.

### FcμR Protects Mice against Bacteria-Induced Sepsis

Having found that FcμR is required for MZB response to LPS, we next sought to investigate the role of FcμR in protecting mice against intravenous bacterial infection. We infected mice with *C. rodentium*, a Gram-negative bacterium that has LPS on its outer membrane. As shown in Figure 6D, *FcμR*^−/−^ mice exhibited significantly accelerated and increased death after the infection compared with WT mice. Along with a previous study demonstrating a critical role for B cells in enhancing early innate immune responses and protecting mice from bacterial sepsis (42), our results suggest that FcμR contributes to B cell-mediated protection against acute bacterial infection.

## Discussion

In this study, we have elucidated a critical role for FcμR in tonic BCR signaling in MZB and in their survival and LPS response *in vivo*. In addition, FcμR is required for antibody production against the T-I Ag NP-LPS and in protection against bacterial sepsis. Along with previous findings (22), these results demonstrate that FcμR is required for the survival and activation of both FOB and MZB and regulates both adaptive and innate immune responses.

While we found reduced MZB population in *FcμR*^−/−^ mice [(22, 25), and current study], Nguyen et al. found reduced proportion of MZB but their absolute numbers were not decreased due to an increase in the total splenic B cells (27). The reason for this discrepancy is unclear but could be due to the difference in the age of mice. We have analyzed mice under 12-week-old, whereas the age of their mice used in the analysis of MZB was not indicated. In addition, we analyzed mice with a global FcμR deficiency whereas they analyzed mice with CD19-Cre-mediated B cell-specific FcμR deletion. Although FcμR is predominantly expressed by B cells (22–27), one cannot exclude the possibility that FcμR is also expressed at low levels by other cell types, which might have affected MZB. Furthermore, CD19 is a part of BCR coreceptor and it has been shown that humans carrying one mutated CD19 have defects in B cell development and activation (43, 44). CD19-Cre-mediated FcμR deletion also resulted in CD19 heterozygosity, which might have some effects on B cell development or function.

While *FcμR*^−/−^ mice have reduced MZB population, mice lacking soluble IgM (Sμ^−/−^), the specific ligand for FcμR, showed a threefold increase in MZB numbers that is normalized by passive administration of polyclonal IgM (45). Similarly, Sμ^−/−^ mice, but not *FcμR*^−/−^ mice, exhibited enhanced B-1 cell development in the peritoneal cavity (46, 47). We have previously shown that FcμR physically associates with surface BCR in normal spleen B cells (33). FcμR therefore binds to both soluble pentameric IgM and membrane BCR. One intriguing possibility is that soluble IgM and BCR compete for the binding to FcμR. In Sμ^−/−^ mice where soluble IgM is absent, FcμR may preferentially associate with BCR, leading to distinct phenotypes in MZB and B-1 development.

We show in this study that FcμR is required for the survival of MZB *in vivo*. Tonic BCR signal is essential for B cell survival. While WT and *FcμR*^−/−^ MZB expressed the same levels of surface IgM BCR, we found reduced levels of pSYK and AKT in *FcμR*^−/−^ MZB compared with WT MZB. SYK phosphorylation and activation occur during the early phase of BCR signaling. Therefore, the reduced SYK phosphorylation in *FcμR*^−/−^ MZB suggests that FcμR is involved in the proximal BCR signaling, possibly upstream of SYK, in MZB. pSYK further activates downstream targets including AKT. Phosphorylated AKT promotes B cell survival by inhibiting FOXO1-mediated expression of pro-apoptotic genes (48). The reduced levels of pSYK and AKT suggest that FcμR promotes MZB survival *in vivo* by enhancing tonic BCR signaling. While we found that pSYK and pAKT levels were not significantly different between WT and *FcμR*^−/−^ FOB, Nguyen et al. found increased pAKT levels in FOB of mice with B cell-specific FcμR deletion when compared with control littermates (27). As is the case for the discrepancy in MZB, differences in the age of mice, global vs. B cell-specific FcμR deficiency, or CD19 haploinsufficiency could have affected the results. Nevertheless, both their study and our previous study (22) revealed increased IgM levels in spleen B cells. In addition, the increases in pAKT levels and cell viability after anti-IgM stimulation were clearly compromised in *FcμR*^−/−^ B cells compared with WT B cells (27), which is consistent with our study (22, 33) showing reduced B cell survival in *FcμR*^−/−^ B cells after BCR crosslinking.

While FcμR is required for the survival of MZB *in vivo* in the absence of exogenous Ag stimulation, we have previously found that FcμR is required for the survival of FOB only after BCR crosslinking. Consistently, *FcμR*^−/−^ mice have reduced MZB but normal FOB population. It has been suggested that MZB frequently recognize self Ag, for example Sm/RNP, which can be released from dead cells. Therefore, even in the absence of exogenous BCR stimulation, MZB may be constantly stimulated by self Ag. As MZB express much higher levels of IgM, but not IgD, BCR than FOB, self Ag stimulation may trigger a sufficiently strong signal in an FcμR-dependent manner. It is therefore conceivable that the “tonic” BCR signaling and the consequent SYK and AKT phosphorylation observed in MZB are actually a result of self Ag stimulation. In this regard, self Ag-stimulated MZB may resemble exogenous Ag-stimulated FOB. Therefore, FcμR may function similarly in FOB and MZB, i.e., promoting B cell survival after BCR stimulation, although the nature of the Ag may be different between FOB and MZB.

Fc receptor deficiency did not result in reduced expression of RBP-J/Notch2, which plays a critical role in the MZB fate determination. Notch2 expression was even slightly elevated in *FcμR*^−/−^ MZB. This result suggests that FcμR is unlikely involved in MZB fate decision or commitment to the MZB lineage. In addition, we found a moderate but statistically significant increase in the levels of IgD and MHC-II. It has been shown that IgD BCR can only be activated by polyvalent Ag (49). Therefore, the elevated IgD levels in *FcμR*^−/−^ MZB might affect their responses to Ag stimulation. MHC class II is normally upregulated in B cells upon activation. The moderate increase of MHC class II in *FcμR*^−/−^ MZB may suggest that MZB in *FcμR*^−/−^ mice might be at a slightly activated status. Alternatively, the elevated IgD, MHC-II, and Notch2 expression could be a result of the increased turnover of MZB observed in *FcμR*^−/−^ mice, possibly to counteract against the reduction of MZB. A similar phenomenon has been observed in mice lacking c-myb where FOB were reduced in the periphery but these FOB exhibited increased turnover and expressed elevated MHC-II (50).

Recently, *FcμR*^−/−^ mice were found to contain increased titters of the anti-Sm/RNP autoantibodies (37). Since antibodies against Sm/RNP are known to be produced by MZB (51), it was speculated that the reduction of MZB cells in *FcμR*^−/−^ mice was caused by the rapid differentiation of MZB into plasma cells. To verify the accelerated plasma cell differentiation of MZB in *FcμR*^−/−^ mice, we have attempted to immunize mice i.v. with Sm/RNP. However, we were unable to find an increased production of α-Sm/RNP antibodies in *FcμR*^−/−^ mice (Figure S4 in Supplementary Material). We think that *FcμR*^−/−^ mice already contain elevated titers of α-Sm/RNP antibodies, which may neutralize the immunized antigen quickly and prevent further production of the antibodies against these Ag. Based on these observations, we propose that FcμR has dual functions: it promotes antibody production by MZB against foreign T-I Ag yet restricts the production of autoantibodies such as α-Sm/RNP.

An unexpected finding is that FcμR is required for MZB to respond to LPS. LPS stimulates TLR4 and triggers survival and activation signals in B cells through MyD88-dependent and -independent pathways. Intriguingly, very recently, it has been reported that TLR4 signaling in B cells requires BCR and SYK (52). Their results suggest that TLR4 signals through two distinct pathways, one *via* BCR and the other *via* MYD88. Based on these findings, the poor response of *FcμR*^−/−^ MZB to LPS may be attributable to the reduced BCR signaling in these cells. We also found reduced antibody production against the type 1 T-I Ag NP-LPS. MZB and B-1 cells are thought to be the major cell types that respond to the T-I Ag. *FcμR*^−/−^ mice have reduced MZB but normal B-1 population, suggesting that the impaired antibody production against NP-LPS in *FcμR*^−/−^ mice is more likely attributable to both the decreased MZB population and their impaired response to LPS. MZB are known to play a central role in capturing and responding to the blood-borne Ag (41). It has also been shown that B cells protect mice against bacterial sepsis by enhancing innate inflammatory cytokine and chemokine production (42). In agreement with this study, we found that *FcμR*^−/−^ mice exhibited accelerated and increased death after intravenous bacterial infection compared with WT mice. We postulate that *FcμR*^−/−^ mice were unable to efficiently capture the blood-borne *C. rodentium* due to reduced MZB population and produce inflammatory cytokines due to the impaired LPS response, resulting in increased death. However, it is also possible that defects in other B cell subpopulation or even in non-B cells might be involved in the accelerated death of *FcμR*^−/−^ mice after i.v. inoculation of *C. rodentium*. In summary, this study revealed an important role for FcμR in the survival and activation of MZB and in protection against bacterial sepsis.

## Ethics Statement

All animal experiments were approved by the Animal Committee of the School of Basic Medical Sciences, Fudan University.

## Author Contributions

JL designed the study, performed the experiments, and contributed to the writing of the manuscript. HZ and JQ analyzed and interpreted data. EX and LZ helped animal experiments. Y-QW, YC, HK, and TT interpreted data and supervised the study. J-YW designed the study, interpreted data, and wrote the manuscript. All authors read and approved the final manuscript.

## Conflict of Interest Statement

The authors declare that the research was conducted in the absence of any commercial or financial relationships that could be construed as a potential conflict of interest.
