# Supplementary material for: Fcµ Receptor Promotes the Survival and Activation of Marginal Zone B Cells and Protects Mice against Bacterial Sepsis
Source: Front Immunol. 2018 Feb 5;9:160. doi: 10.3389/fimmu.2018.00160 (PMC5807594; doi:10.3389/fimmu.2018.00160)
Supplement: Supplementary file 1 [file Table_1.DOCX]

| Antigen | Conjugation | Source | Cat-Number | Isotype （clone） | Company |
| --- | --- | --- | --- | --- | --- |
| CD19 | APC | Rat | 550992 | IgG2a (1D3) | BD Bioscience |
| CD19 | FITC | Rat | 553785 | IgG2a (1D3) | BD Bioscience |
| CD21 | PE-Cy7 | Rat | 123420 | IgG2a (7E9) | Biolegend |
| CD21 | FITC | Rat | 553818 | IgG2b (7G6) | BD Bioscience |
| CD23 | PE-Cy7 | Rat | 101613 | IgG2a (B3B4) | Biolegend |
| CD23 | PE | Rat | 553139 | IgG2a (B3B4) | BD Bioscience |
| CD40 | APC | Rat | 558695 | IgG2a (3/23) | BD Bioscience |
| CD80 | PE | Hamster | 12-0801-81 | IgG (16-10A1) | eBioscience |
| CD86 | APC | Rat | 561964 | IgG2a (GL1) | BD Bioscience |
| CD93/AA4.1 | APC | Rat | 17-5892-81 | IgG2b (AA4.1) | eBioscience |
| CD93/AA4.1 | Biotin | Rat | 13-5892-81 | IgG2b (AA4.1) | eBioscience |
| CD95/Fas | PE | Mouse | 12-0951-81 | IgG1 (15A7) | eBioscience |
| CD138 | APC | Rat | 558626 | IgG2a (281-2) | BD Bioscience |
| CD169/MOMA-1 | Biotin | Rat | MCA947GA | IgG2a | BIO-RAD |
| B220 | APC | Rat | 103212 | IgG2a (RA3-6B2) | Biolegend |
| B220 | FITC | Rat | 553088 | IgG2a (RA3-6B2) | BD Bioscience |
| B220 | APC-Cy7 | Rat | 552094 | IgG2a (RA3-6B2) | BD Bioscience |
| IgM | PE | Mouse | 553521 | IgG1 (AF6-78) | BD Bioscience |
| IgM | Percp-Cy5.5 | Rat | 550881 | IgG2a (R6-60.2) | BD Bioscience |
| IgD | PE | Rat | 558597 | IgG2a (11-26c.2a) | BD Bioscience |
| MHC-II | PE | Mouse | 12-5320-82 | IgG2a (AF6-120.1) | eBioscience |
| TLR4 | PE | Rat | SC-13591 | IgG2a (MTS510) | Santa Cruz Bio |
| Syk (pY348) | PE | Mouse | 558529 | IgG1 (I120-722) | BD Bioscience |
| Akt (pS473) | Alexa Fluor647 | Mouse | 560343 | IgG1 (M89-61) | BD Bioscience |
| Notch2 | PE | Hamster | 130707 | IgG (HMN2-35) | Biolegend |
| Caspase-3 | PE | Rabbit | 51-68655X | IgG(C92-605) | BD Bioscience |
| Streptavidin | PE |  | 554061 |  | BD Bioscience |
| EdU |  |  | C10424 |  | Invitrogen |
| NP-LPS |  |  | N-5065-5 |  | Biosearch Technologies |
| 7-AAD |  |  | 00-6993-50 |  | eBioscience |
| LPS |  |  | L3012 | O111:B4 | Sigma |
| Sm/RNP antigen |  |  | ATR01-02 |  | AROTEC Diagnostics Limited |

Supplemental Table 1. List of reagents used in the current study
