# Supplementary material for: Fcµ Receptor Promotes the Survival and Activation of Marginal Zone B Cells and Protects Mice against Bacterial Sepsis
Source: Front Immunol. 2018 Feb 5;9:160. doi: 10.3389/fimmu.2018.00160 (PMC5807594; doi:10.3389/fimmu.2018.00160)
Supplement: Supplementary file 2 [file Presentation_1.PDF]

Supplemental Figure 1

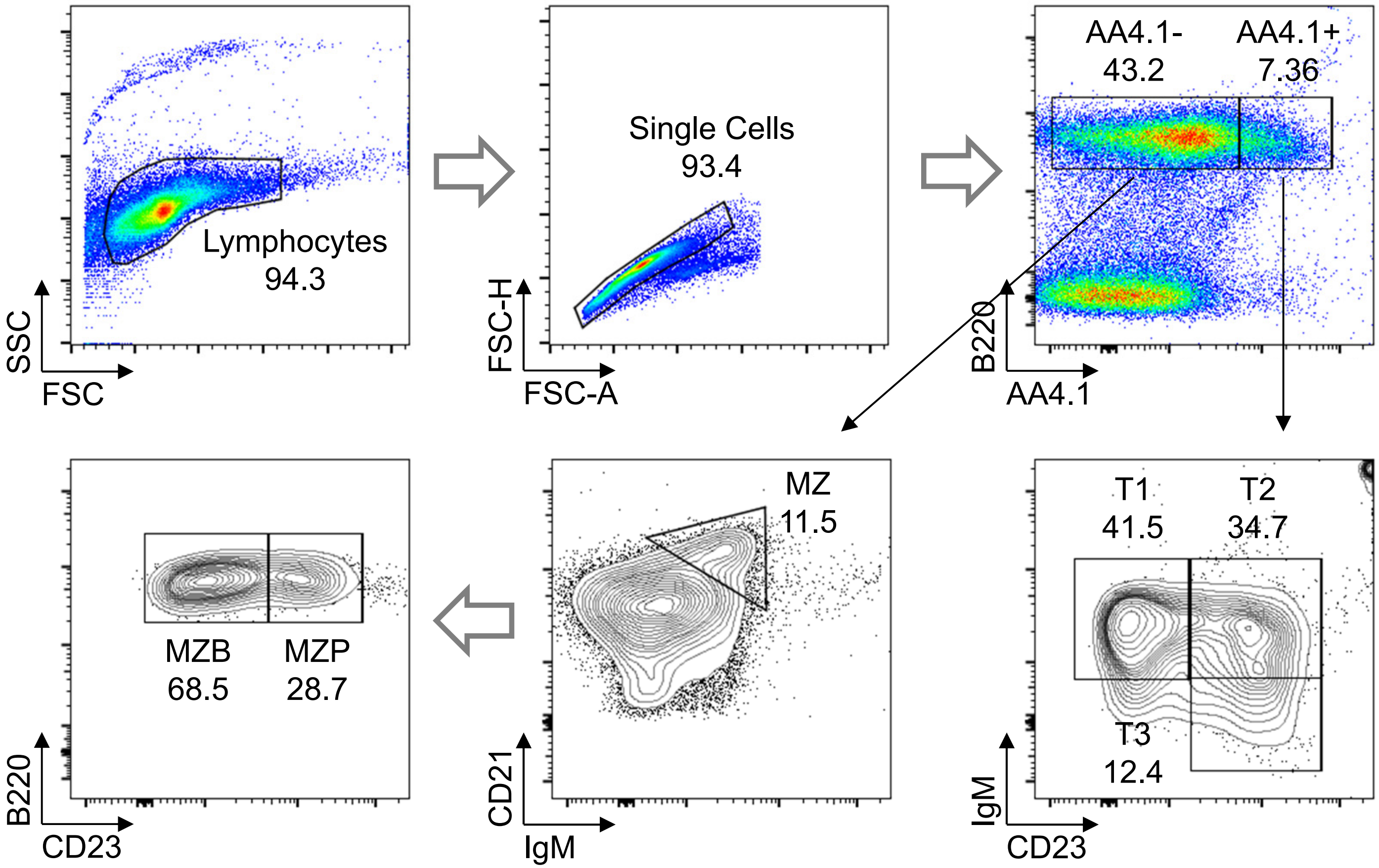

Fig. S1 Gating strategy for the analysis of MZB cell development.

Supplemental Figure 2

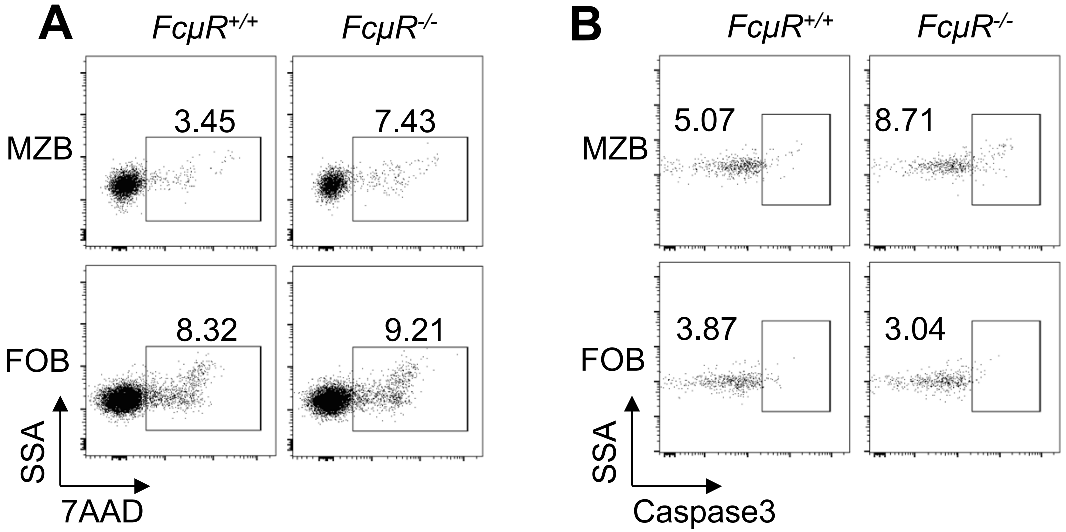

Fig. S2 Representative FACS profiles for the analysis of cell death in freshly isolated MZB and FOB. Splenocytes were stained with α-CD23 and α-CD21 in the presence of 7AAD as described in Materials and Methods and analyzed for $7AAD^{+}$ cells in gated $CD21^{high}CD23^{low}$ (MZB) and $CD21^{low}CD23^{high}$ (FOB) population.

Supplemental Figure 3

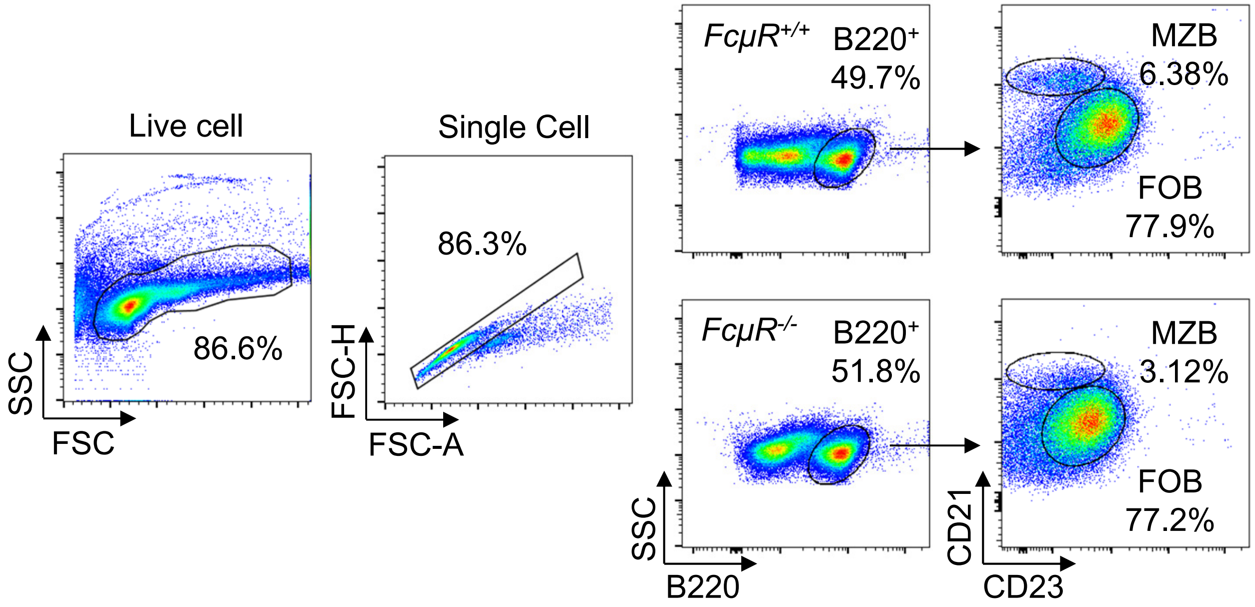

Fig. S3 Gating strategy for the analysis of pSYK and pAKT expression in MZB and FOB. Live and single splenocytes were analyzed for their B220, CD21 and CD23 expression. pSYK and pAKT levels in gated $B220^{+}CD21^{high}CD23^{low}$ (MZB) and $B220^{+}CD21^{low}CD23^{high}$ (FOB) are shown in Fig. 5A and B.

Supplemental Figure 4

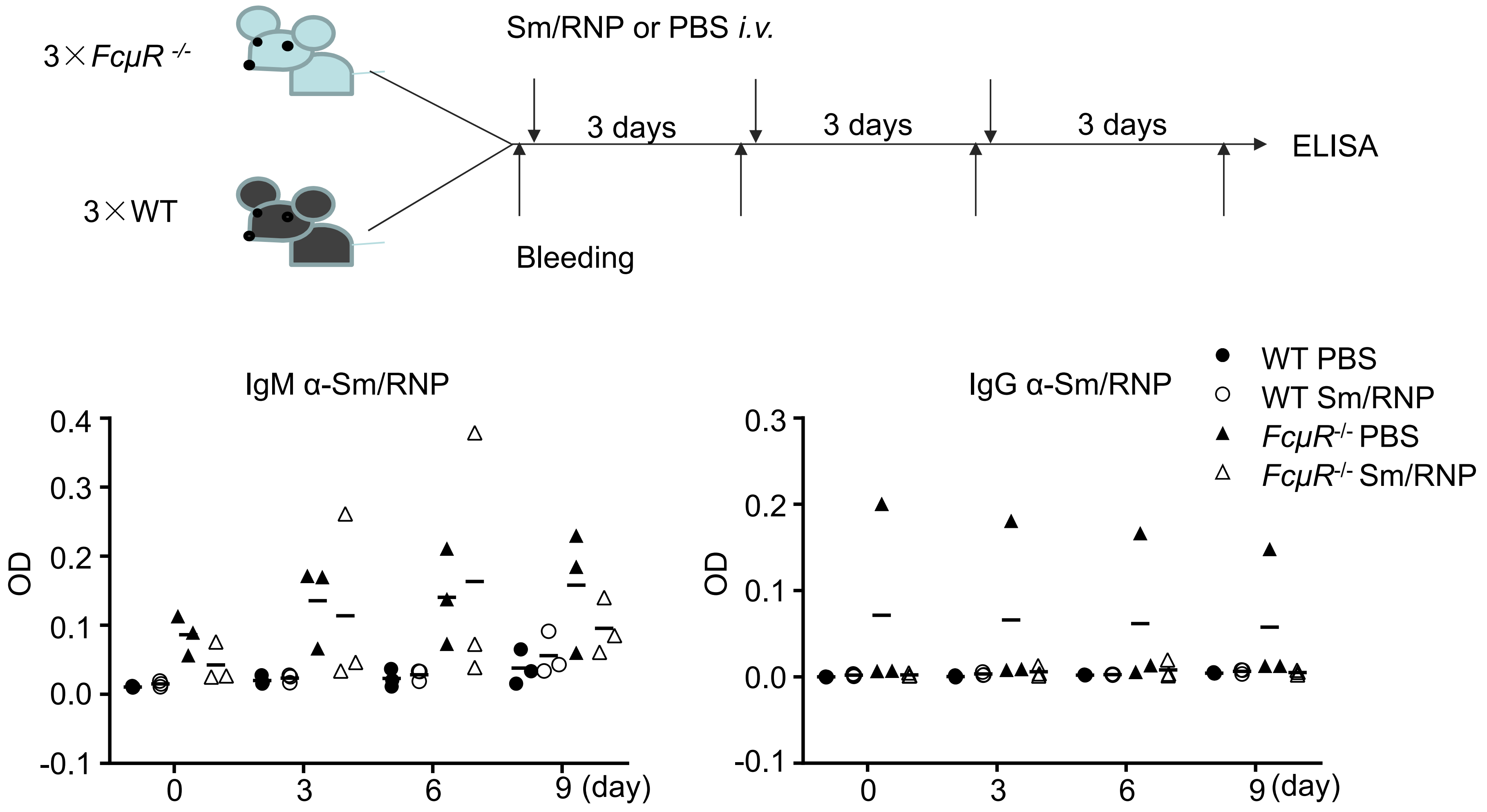

Fig. S4 Induction of α-Sm/RNP antibodies by immunization with Sm/RNP *i.v.*. Three pairs of WT and *FcμR*-/- mice (10 wk-old) were immunized with 25μg of Sm/RNP or PBS as a control and bled every 3 days as indicated above. IgM and IgG α-Sm/RNP antibodies were measured by ELISA.
